# Whose Knowledge? Examining the Relationship between the Traditional Medicine Sector and Environmental Conservation Using a Stakeholder Analysis: Perceptions on Warwick Herb Market Durban South Africa

**DOI:** 10.3390/ijerph191911900

**Published:** 2022-09-21

**Authors:** Nontando N. Xaba, S’phumelele L. Nkomo, Kirona Harrypersad

**Affiliations:** School of Agricultural, Earth and Environmental Sciences, University of KwaZulu Natal, Durban 4001, South Africa

**Keywords:** stakeholders, compliance, law enforcement, biodiversity conservation, political ecology

## Abstract

The South African traditional medicine sector is estimated to accommodate millions of citizens, despite it being informal. The existence of such a healthcare system embodies the dual system of both primary and traditional healthcare, with some preferring one and others utilising both systems. The gathering, harvesting, and selling of medicinal plant and animal species have inevitable environmental effects. The paradox between biodiversity conservation and livelihood sustenance is eminent in South Africa’s contemporary environmental legislation. The purpose of the study was to highlight and examine the dynamics between prominent stakeholders involved in biodiversity conservation and the traditional medicine sector. The stakeholder analysis and political ecology approach were adopted and applied respectively to guide the study. The study was conducted in 2020 and a questionnaire was used to capture the realities and experiences of prominent stakeholders in the biodiversity sector. Common legal mandates such as the Convention on Biological Diversity (CBD); Convention on International Trade in Endangered Species of Wild Fauna and Flora (CITES); the National Environmental Management Biodiversity Act No. 10 of 2004; Threatened or Protected Species (TOPS) regulations; and the KwaZulu-Natal Nature Conservation Ordinance 15 of 1974 are used to control and enforce legislation by biodiversity stakeholders. The main findings of the study are as follows: (a) Traditional Health Practitioners (THPs) do not have adequate training and knowledge on the environmental and legal aspects of their system; (b) Biodiversity stakeholders are treated with violence and hostility when they attempt to enforce legal mandates at the Warwick Herb Market; (c) There is a significant gap in communication and co-operation between municipal officials and biodiversity stakeholders. There is evidently, a need for environmental educational initiatives and improved methods of enforcement and communication between biodiversity stakeholders, municipal officials and THPs.

## 1. Introduction

Traditional medicine co-exists with the modern health care sector and plays an important role in the lives of South Africans. Peterson et al. (2017) estimate that the traditional medicine sector accommodates about 27 million South African citizens [1]. Mainstream literature argues that modernisation and advancement of healthcare will reduce the use of traditional medicine [2,3,4]. Despite the availability of primary healthcare, traditional medicine is still regarded as an important part of the lives of many South African citizens [1]. Since the early 1990s, the number of THPs was estimated around 350,000. In 2009, the ratio between THPs and the population was 500:100,000, with 77:100,000 between medical doctors and people [5]. In 1998, there was an estimated six million consumers of traditional medicine in KwaZulu-Natal [6]. Due to the lack of statistical evidence, one can assume the figures have gradually declined due to the modern trends associated with rural-urban migration and the prevalence of allopathic healthcare facilities. Total revenue generated from primary and secondary indigenous resources was approximately R482 million in 2011, with R322 million exported [7].

As the population grows the demand for traditional medicine in South Africa continues to escalate annually. Subsequently, the demand for traditional medicine has contributed to the growth of the traditional medicine industry with an estimated 50 to 100 informal market enterprises and the subsequent job creation [8].

Whilst researchers note the importance of traditional medicine, environmental impacts of traditional medicine, both on plants and animals, cannot be ignored [9,10]. Estimated to be worth R2.9 billion annually, environmental consequences to the traditional medicine trade are inevitable [11,12,13]. The increase in the demand for traditional medicine will result in detrimental effects on South Africa’s rich biodiversity and communities relying on the environment for their livelihoods [14]. Therefore, there is a need for a multi-stakeholder approach to understanding the nexus between traditional medicine and environmental management and enforcement.

## 2. South African Environmental Governance: Biodiversity Conservation and Traditional Medicine

Traditional medicine in a democratic South Africa signifies the existence of a dual healthcare system, with segments of the population utilising both whilst some with stringent dependence on the former [15]. There is, therefore, a need for strong governance and facilitation around the trading of medicinal flora and fauna, subsequently the traditional medicine sector. The need for strong governance and facilitation is due to human activities such as habitat destruction and unsustainable harvesting practices that will lead to species extinction, therefore, threatening the availability of medicinal plants [16]. In South Africa, government institutions, environmental groups, and non-governmental organisations, with the sole purpose of promoting biodiversity conservation, sustainable development, and livelihood sustenance, facilitate environmental management.

Environmental compliance and enforcement are a vital necessity for effective and sustainable environmental management. Environmental compliance and enforcement are used interchangeably as they both denote the processes involved in ensuring that groups, individuals, or communities comply with environmental legislation [17]. The International Network for Environmental Compliance and Enforcement (INECE) stipulate essential requirements for effective compliance and enforcement programmes, five of which are contextualised in Table 1.

From a South African context, there has been substantial progress in ensuring that environmental legislation is enforced [18]. Environmental bodies and institutions are principally mandated by legislation to ensure that the trading of medicinal flora and fauna is permittable under sustainable grounds. The challenge comes with compliance from traditional medicine traders, who are predominately profit-driven and subsequently exploit the system. It is, therefore, challenging to balance the paradox of livelihood sustenance with environmental conservation. Table 1, therefore, highlights the importance of promoting compliance through education and awareness initiatives. Education and awareness have the potential to change mindsets and possibly mitigate and alleviate the threats to the environment. In addition to education and awareness, there is a need for stringent enforcement measures for violators. Measures such as suspension of trading permits and issuing of warnings are vital. Once enforcement has been applied, there is a need for constant evaluation and assessment of the applied measures [19].

**Table 1:** Contextualisation of INECE’S standard requirement with South Africa’s Traditional Medicine Sector.

INECE’s Standard Requirements	South African Traditional Medicine Sector
Creating enforceable requirements	Establishing policies that fit the scope of the traditional medicine sector.
Knowing who is subject to the requirements	Identifying groups/individuals that are most likely to contravene environmental legislation.
Promoting compliance in the regulated community	Awareness programmes aimed at educating THPs about the environmental impacts of wildlife harvesting and trade. Promotion of compliance through the provision of incentives. E.g., Water tanks; Irrigation systems; School funding.
Responding to violations	Implementation of enforcement measures for violators. E.g., Suspension of trading permits. Prosecution; Fines.
Evaluating the success of the compliance and enforcement programme	Monthly monitoring of compliance. Evaluation of existing programmes and adjustments to fit the environmental and socioeconomic scope of the communities affected.

INECE’S standard requirements sourced from [20] (p. 44).

Environmental law enforcement refers to the actions that designated officials take to achieve compliance within a group of individuals or communities and to cease situations where compliance is not present [17]. In South Africa, the orthodox mechanism of law enforcement is arrest and criminal prosecution. Over the years the government has made numerous attempts to improve environmental law enforcement in the conservation sector, including the formulation of permits and community-based mechanisms aimed at improving compliance [21]. Sachs et al. (2009) (p. 45) argue that:

“*In South Africa, many of the prescribed environmental standards are outdated and accordingly the traditional mechanisms used to regulate* *behaviour and ensure compliance therewith, such as environmental permits with associated conditions, have on occasion proven inappropriate*”.

[22]

It is therefore crucial that mechanisms of law enforcement are improved to ensure that contemporary environmental issues are mitigated and prevented. Community-based mechanisms are most effective when there are incentives involved. A community is most likely to comply with environmental legislation when there are benefits involved. The theory of contingent consent is used to explain the relationship between compliance with environmental legislation and the incentives provided. According to this theory, “States secure the compliance of their people when they strike a policy bargain the citizenry perceives as just” [22] (p. 1). In a democratic South Africa, the most vulnerable communities are heavily dependent on the incentives provided by the government [23]. Government and conservation institutions incentivize basic needs in return for environmental compliance, therefore potentially diminishing the need for stringent environmental law enforcement mechanisms [24]. Community-based conservation programmes initiated after 1994 engage both South African conservation agencies and traditional healers [25].

Amongst the many incentives, the implementation of development projects; conservation grants; and employment opportunities in conservation for individuals from disadvantaged communities are examples of how the government implements incentives to promote conservation [26]. Makunga et al. (2008) (p. 367) elaborate that “participatory schemes which involve the conservationists and user groups of medicinal plants (local communities) are beginning to consider gender, cultural, ethnic and other social values in managing biodiversity” [25]. The incentives-based approach is not exempted from problems. According to Spiteri and Nepalz (2006), in some cases, the incentive-based approach, is unable to create unity within communities, therefore, progressing the challenges to biodiversity conservation [24]. Hence, the role of legal mandates for biodiversity conservation in South Africa remains paramount.

## 3. Legal Mandates for Biodiversity Conservation: Traditional Medicine Sector

The effectiveness of a country’s legislation is reflected in the level of compliance by the country and its citizens. South Africa has a solid Constitution that covers all facets of governance, including environmental rights that advocate for citizens to have the right to “An environment that is not harmful to their health or wellbeing” [27] (Section 24, p. 11). The Department of Environment, Forestry and Fisheries (DEFF) is the primary custodian for South Africa’s natural environment. From a traditional medicine perspective, DEFF facilitates the biodiversity economy through eco-tourism, bioprospecting and biodiversity conservation services and its related policies. Bioprospecting is regulated through legislation that emanates from the Nagoya Protocol of 2010, on access and benefit-sharing of genetic resources with the emphasis on fair and equitable sharing of benefits arising from their utilisation [28]. South Africa is also obligated to international environmental mandates through treaties such as the Convention on Biological Diversity (CBD) and the Convention on International Trade in Endangered Species of Wild Fauna and Flora (CITES) amongst many others. The obligation to these international standards ensures that environmental governance is effective and sustains the livelihoods of the citizens. 

Amongst the treaties, the CBD is the most relevant to the traditional medicine sector and subsequent legislation. South Africa signed the CBD in 1993 and has been a member state of this convention since 1995 [29]. The primary principle of the CBD is the importance of, and need to promote international, regional and global cooperation amongst countries and intergovernmental sectors for the conservation of biological diversity and the sustainable use of its components [29]. Two decades ago, the international illegal commercial trade in wildlife amounted to US $5 to 50 billion annually, an alarming figure that calls for strict legal control [18]. In parallel, CITES works through the subjection of international trade in specimens of selected species to certain controls [30]. South Africa falls under category 2 of the quality of National CITES legislation in African countries that are situated in the south of the Sahara. Category 2 means that South Africa has legislation which is believed generally not to meet all requirements for the implementation of CITES [31]. China is said to be the dominant source of global demand for wildlife products with South Africans actively supplying the market [32].

In CITES plant categories, traditional medicine falls under the Non-timber Forest Products (NTFP). Traditional medicine is considered to be the largest within this category. Schippmann et al. (2006) estimate that 50,000 to 70,000 medicinal plants are used worldwide [33]. Current figures on the number of medicinal plants used worldwide are however unavailable [33]. The deficiency in figures may be attributed to the informal and unregulated nature of the traditional medicine trade. In addition, the scarcity of information on medicinal plant markets reflects the level of understanding of the NTFP markets in general, even in economically advanced countries [34]. Overharvesting, habitat loss, climate change and international trade contribute towards the estimated 15,000 threatened medicinal plant species [33,34]. It is therefore crucial that conservation strategies are improved and applicable to the complex and demanding traditional medicine trade.

In South Africa, environmental law enforcement is facilitated and administrated by the Environmental Management Inspectorate (EMI) commonly known as the “green scorpions” [35]. The EMI was formulated through the Amendment of the National Environmental Management Act (NEMA) in 2005. Paterson and Kotze’ (2009) articulate that the NEMA Act provides for the EMI which is intended for the appointment of government officials to monitor and enforce environmental legislation [36]. The EMI is eligible to collaborate with other bodies of enforcement such as the South African Police Service, towards effective enforcement and monitoring.

Environmental monitoring and enforcement may be designated to EMI, but overall prosecution is the responsibility of the National Prosecuting Authority [22,35,37]. The duties and mandates of EMI are based on their grades. A compliance notice is a primary tool used by EMI to ensure environmental law enforcement and compliance is accomplished. Compliance notices may only be issued by EMIs ranging from grade 1 to grade 3. An EMI may only issue a compliance notice under the Section 31L (1) of the NEMA Act which stipulates that “if there are reasonable grounds for believing that a person has not complied with a provision of the law for which that inspector has been designated” [37] (p. 14). It is pivotal for South African enforcement to be monitored, in order to identify gaps and improvements in South African environmental legislation.

Within these legal instruments, there are compatible concepts that are vital for the understanding of various categories in which stakeholders can enforce environmental legislation (Table 2).

In addition to Convention on Biological Diversity (CBD), CITES, the National Environmental Management: Biodiversity Act (NEMBA) and Threatened or Protected Species (TOPS) regulations are mandatory for the control and conservation of flora and fauna and South Africa.

**National Environmental Management: Biodiversity Act, 2004 (Act No. 10 of 2004): Threatened or Protected Species regulations.**

Under Section 97 of the NEMBA, there are listed regulations relating to the TOPS [38]. The purpose of regulations as listed under Section 2:

(a) Further regulate the permit system set out in chapter 7 of the Biodiversity Act insofar that system applies to restricted activities involving specimens of listed threatened or protected species;
(b) provide for the prohibition of specific restricted activities involving specific listed threatened or protected species;
(c) Provide for the protection of wild populations of listed threatened species; and
(d) provide for the composition and operating procedure of the Scientific Authority.

[38] (pp. 25–26)

From a traditional medicine perspective, these regulations stipulate the necessity of the permit system as well as the operations and functions of the scientific authority. These regulations further stipulate the vitality of protecting threatened species and overall biodiversity conservation. Environmental management inspectors are therefore mandated to enforce and carry out their functions based on the NEMBA/TOPS regulations.

The evaluation of primary legal mandates used for environmental law enforcement is mandatory to understand the roles and functions of the involved stakeholders. The CBD; CITES; NEMBA No. 10 of 2004, TOPS regulations and the KwaZulu-Natal Ordinance 15 of 1974 are amongst the legal instruments used by stakeholders to enforce biodiversity conservation within the traditional medicine sector. Furthermore, stakeholder perceptions and involvement are analysed thematically for an understanding of the realities and challenges around the regulation of the traditional medicine sector whilst enforcing contemporary environmental legislation. Thematic analysis is necessary for the identification of common perceptions relating to the subject matter.

Against this background, this paper intends to examine the relationship between the traditional medicinal sector and environmental management and enforcement at the Warwick Herb Market, Durban, South Africa. By using stakeholder analysis and political ecology as conceptual frameworks, this paper tries to map and analyse the interests, practices, and power relations of various stakeholders. Stakeholder analysis is one of the important frameworks that has been used extensively in environmental and natural resource management [40]. The paper extends the stakeholder’s analysis by incorporating political ecology to examine power relations and the politics of knowledge production among multiple stakeholders. These are stakeholders involved in biodiversity conservation and subsequently associated with the interaction of traditional medicine and environmental management.

## 4. Conceptual Framework: Stakeholder Analysis and Political Ecology 

### 4.1. Stakeholder Analysis

Stakeholder analysis is a method used to identify individuals that have power and influence pertaining to a subject topic or policy. Brugha and Varvasovszky (2000) explain that stakeholder analysis seeks to understand the factors involved in decision-making and the implementation of projects or policies [41]. These factors include behaviour, intentions, and resources available. Vogler et al., 2017 further describe stakeholder analysis as a range of techniques used to plan, identify, and assess viewpoints of prominent individuals, groups or institutions on a project or proposed activity [40]. In this regard, stakeholder analysis, therefore, allows the researcher to link various stakeholders that relate to their study in order to analyse how they connect to influence the subject matter [40,41,42]. For example, the stakeholders involved in biodiversity conservation are identified and analysed according to their power and influence on the implementation and enforcement of conservation policies [43]. Once identified, the stakeholder method helps to determine the roles played by the stakeholders as well as the factors influencing the subject or policy.

Conservation in South Africa is facilitated by a variety of institutions, both governmental and private. The mandate is biodiversity conservation, yet the institutions are various and consist of subsequent stakeholders [40]. In this regard, stakeholders may have varying roles (Biodiversity conservation officers; community liaison officers; environmental stewardship managers; environmental educators), but a common mandate (environmental management). Stakeholder analysis, therefore, helps to contextualise how these varying roles complement each other for the implementation of their common mandate.

The variety of roles within stakeholders helps the researcher to identify the level of power and influence of stakeholders. Stakeholders are not only those employed by institutions, but stakeholders also constitute members of the public. Vogler et al., 2017 refer to stakeholders such as members of the public as “hidden stakeholders” [40]. Hidden stakeholders are those directly involved in the subject or policy being implemented or planned. THPs are stakeholders as they are the ones specifically involved in the harvesting and selling of medicinal plants. Law enforcement officers are the stakeholders that ensure that THPs comply with the regulations set by the government and related institutions, which also constitute stakeholders.

The stakeholder theory is said to be introduced by Edward Freeman in 1984 [44]. Schilling (2000) contests this in his argument that the inception of the stakeholder concept was the work of Follet in the year 1918 [45]. Literature, however, associates the stakeholder theory with Freeman. From a business management perspective, the concept of ‘stakeholder’ was originally defined as “those groups without whose support the organisation would cease to exist” [46] (p. 31). The context of the stakeholder theory has not changed, even with the diversity of paradigms and authors, the common notion of stakeholder is power and influence resulting in change [47,48,49,50].

The variance in the definition is dependent on the approach, business management literature will have a different approach to environmental management literature with reference to the stakeholder theory. Ackerman and Eden (1998) view stakeholders from an exclusivity perspective, hence they define stakeholders as those individuals belonging to the organisation that has the power to directly affect the outcome of an organisation [49].

In this regard, without these stakeholders, there is no outcome or growth of the organisation. Contrastingly, some scholars adopt an all-inclusive approach, where stakeholders include even those not belonging to the organisation have the power and potential to change the outcome and decisions taken [48,50]. The environmental management approach to stakeholders is appropriate for the study of THPs and their interaction with environmental legislative bodies and institutions, as they are included and acknowledged in the decisions and change implemented.

**Figure 1:**
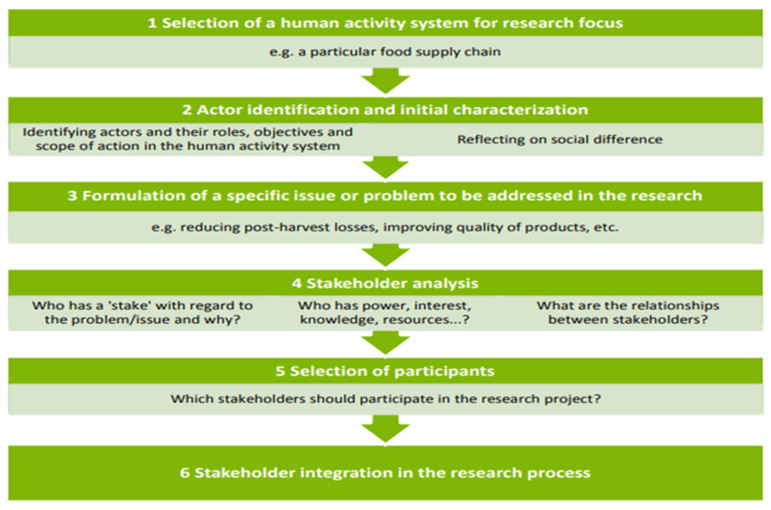
Diagrammatic representation of the steps applied in the stakeholder analysis method for environmental management projects.

Stakeholders in the environmental management field, are faced with the challenge of ensuring that livelihoods are maintained whilst conserving and protecting vital resources of the natural environment. To combat contemporary environmental problems, stakeholder involvement is a vital necessity on national and international scales [42]. The primary role of stakeholders is to participate in the decision-making involved in environmental governance and policy formulation. Decision-making ought to be flexible and transparent in order to accommodate the dynamic nature of the environment and diverse societies [42,51]. Stakeholder analysis is vital in the environmental management field as it determines the key players involved in decision-making and policy formulation pertaining to the sustainability of the environment. In Figure 1, Lelea et al. (2014) explain stakeholder analysis, in order to understand the steps taken when applying the method for environmental management projects [43].

The steps highlighted in Figure 1, are crucial for the effectiveness of the stakeholder analysis method for environmental management projects. Stakeholder Analysis helps the researcher to identify the roles and potential contributions of stakeholders for the project conducted. Post stakeholder identification, the project is then planned according to the stakeholder’s contributions and powers towards the project implementation. It is pivotal to conduct stakeholder analysis prior to the data collection phase, as the gaps and limitations of the subject matter are identified. For example, Figure 1, highlights that stakeholder analysis allows the researcher to identify those that have a stake regarding the problem or issue. For example, stakeholders from Ezemvelo KZN Wildlife are crucial for the issue of non-compliance by THPs. In this regard, stakeholder analysis prevents the researcher from interviewing stakeholders that are irrelevant and have minimal contributions to eliminating the problem [42,43,44].

**Figure 2:**
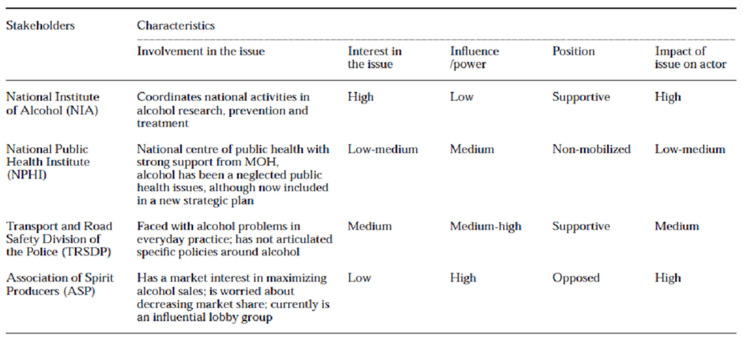
Stakeholder characteristics around the development of a comprehensive national alcohol policy. Source: [53] (p. 342) A stakeholder analysis.

**Figure 3:**
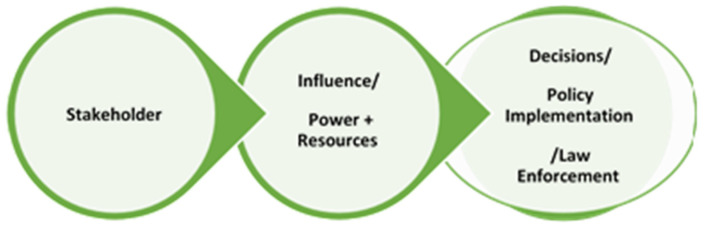
Life cycle of stakeholder role in environmental management projects.

In a study conducted by Brugha and Varvasovszky (2000), a stakeholder analysis was conducted for alcohol policy development in Hungary [40] (Figure 2). The involvement and interest of stakeholders in a project or policy have an impact on the issue (Figure 3). Stakeholder interest can be high but have a low influence or power on the subject matter or project. It is pivotal for the researcher to determine which stakeholder has greater influence as they are the ones that determine the outcome of the matter being dealt with [52]. Typically, THPs can have a high interest in the conservation of medicinal plants but contrastingly the government has a greater influence on whether these plants are conserved or imported for the global cosmetic market. In this regard, stakeholder power and influence determine decision-making, as illustrated in Figure 3.

### 4.2. Political Ecology

Contrary to stakeholder analysis, political ecology illustrates the political dynamics of environmental narratives [54]. Frank Thone is said to be the pioneer of the political ecology concept, followed by Eric Wolf in 1972 [55]. Bridge et al., 2018 contest that, political ecology’s roots are deeper and broader than commonly acknowledged, and that the field is a manifestation of rethinking the relationship between nature and society [56]. The “origin myth” articulates that political ecology emerged from cultural ecology and was initiated by Piers Blaikie, Harold Brookfield, and Michael Watts [56]. Essentially, the political ecology framework highlights key players and how their political clout influences decisions and policies pertaining to the environment. In a nutshell, “Political ecology combines the concerns of ecology and a broadly defined political economy. Together this encompasses the constantly shifting dialectic between society and land-based resources, and also within classes and groups within society itself” [57].

Bryant (1998) articulates those political dynamics and struggles over resources are the essence of the political ecology framework [58]. In this regard, the political ecology concept is ambiguous and varies across disciplines. In his Critical Introductions to Geography, Robbins (2011) provides definitions of the political ecology concept across various disciplines and periods [55] (Table 3). There has been a shift in emphasis on the political ecology concept. The varying emphasis aligns with the transition of societal periods and consequential environmental dynamics.

In the late 1970′s Cockburn and Ridgeway (1979), focus more on the industrial factors that contribute to environmental degradation [59]. This signifies a political economy approach to the nature and social context of political ecology. The concept then transcends to a more societal approach as Hempel (1996) articulates it as a societal reaction to the exploitation of the environment and subsequent decline of resources. Stott and Sullivan (2000) provide a more suitable approach, as they define political ecology as a framework that illustrates the political dynamics of environmental narratives [60]. In this regard, they focus on political circumstances that force people to exploit the environment in the absence of alternative possibilities. The approach used by Stott and Sullivan (2000) is relevant to the dynamics involved in the traditional medicine sector and its nexus to conservation policies.

**Table 3:** Definitions of Political Ecology across discipline, periods, and contexts. Source: [61] (p. 15).

Author/s	Definition	Context
Cockburn and Ridgeway (1979) [59]	“A way of describing the intentions of radical movements in the United States, in Western Europe and other advanced industrial countries” (p. 3).	Attributes environmental degradation to industrial and political activities. Adopt a political economy approach.
Greenberg and Park (1994) [62]	The linkage of the distribution of power with productive activity and ecological analysis.	Adopts a political economy approach. Emphasises bio-environmental relationships.
Peet and Watts (1996) [63]	A confluence between ecologically rooted social science and the principles of political economy.	Broadens environmental issues into a movement for livelihood entitlements and social justice.
Hempel (1996) [64]	Concerned with the political consequences of environmental change (p. 150).	Explores and explains community level and regional political action in the global sphere, in response to local and regional degradation and scarcity (Robbins, 2011: 15).
Watts (2000) [65]	To understand the complex relations between nature and society through a careful analysis of the forms of access and control over resources and their implications for environmental health and sustainable livelihoods (p. 257).	Explains environmental conflict especially in terms of struggles over “knowledge, power and practice” and “politics, justice and governance” (p. 257).
Stott and Sullivan (2000) [60]	Identified the political circumstances that forced people into activities which caused environmental degradation in the absence of alternative possibilities (p. 4).	Illustrates the political dynamics of environmental narratives (p. 5).

## 5. Materials and Methods

### 5.1. Warwick Herb Market

Being one of the nine markets of Warwick, the herb market is one of the city’s culturally diverse and vibrant spaces with a unique history. The market is said to be one of the largest in South Africa and represents a key aspect of the Zulu culture [66]. Located in Bertha Mkhize Street (formerly known as Victoria Street) it is conjoined to the Warwick Junction, a transport interchange that provides access to buses and trains for daily commuters [67]. The Warwick herb market functions within the context of the Inner Thekwini Renewal and Urban Management Program (ITRUMP) [68]. ITRUMP is part of eThekwini Municipality’s initiatives towards sustainability, urban development, and management of the informal economy.

Customers approach the herb sellers and traditional healers with their complaints and the healers supply them with medicine accordingly [69]. To date, there are no exact figures of THPs that occupy the Warwick Herb Market. Subsequently, the Warwick Herb market creates a constant buzz in the city as well as the growth of the informal economy.

“*To consolidate the laws relating to nature conservation and to provide for matters incidental thereto, enacted by the provincial council of KwaZulu-Natal*”.

(United Nations Office on Drugs and Crime, 1974) [70]

The KZN Nature Conservation Ordinance No. 15 of 1974 is amongst the primary legal mandates used to enforce biodiversity conservation in KwaZulu-Natal [70]. It stipulates a broad range of regulations that guide the enforcement and compliance of sectors that use the natural environment. The traditional medicine sector is one that utilises a variety of flora and fauna. Stakeholders are therefore mandated to enforce according to the regulations stipulated in the KwaZulu-Natal Nature Conservation Ordinance No. 15 of 1974 [70]. Table 4 depicts the sections and chapters relevant to the enforcement of the Nature Conservation Ordinance No. 15 of the 1974 legislature [70].

### 5.2. Research Design

The study adopts a mixed-methods approach. Mixed methods research involves the collection, analysis, and interpretation of both quantitative and qualitative data in a single study [71]. The study applies mixed methods through the utilisation of questionnaires for formal interviews. From a quantitative aspect, the questionnaire is used to capture, and code data collected through interviewing the relevant stakeholders. The coded themes are vital for the analysis of the stakeholder’s responses. From a qualitative aspect, the recorded responses are transcribed and categorised under the established themes. Caruth (2013) (p. 112) highlights the benefits of a mixed methods approach:

“*It offers richer insights into the phenomenon being studied and allows the capturing of information that might be missed by utilising only one research design, enhances the body of knowledge, and generates more questions of interest for future studies that can handle a wider range of research questions because the researcher is not limited to one research design*”.

[72]

It can then be argued that adopting the mixed methods approach is relevant and necessary for a study of this nature. The quantitative aspect contributes to the enhancement and narrowing of the qualitative gaps.

### 5.3. Sampling Method

Purposive sampling was used to identify the stakeholders relevant to the study. Purposive sampling involves the selection of stakeholders based on their knowledge and expertise. Purposive sampling is deliberate in the manner that the participants (stakeholders) are selected based on their knowledge and potential contribution to the fulfilment of the study’s objectives [73].

The stakeholders selected for this paper, were those individuals involved in corporate government institutions who are involved either in the formulation and enforcement of environmental legislation; those who monitor and facilitate biodiversity conservation and related practices: and individuals who work closely with THPs whilst advocating the environmental management and sustainable development agenda. Amongst these organisations, Ezemvelo KZN Wildlife, The Department of Environment, Forestry and Fisheries (DEFF), eThekwini Municipality (eThekwini THPs Executive Committee) and the Department of Health were selected and interviewed on the organisational roles and perceptions of the interaction between traditional healthcare and contemporary environmental legislation.

### 5.4. Stakeholder Analysis and Mapping

Stakeholder analysis and mapping were vital for the identification and classification of stakeholders based on their interests, influence, and contribution toward the successful completion of the project. Table 5 illustrates the primary stakeholders that were selected for the study. Each stakeholder represented an organisation and its interests, powers, and practices. The organisations represented in Table 5 are numerically categorised to avoid subjective conclusions and conflict. The names of the organisations are only known by the researcher and the relevant institution. There were a variety of interests and roles across the selected stakeholders, thus a diverse and substantial amount of data was received. The diversity of interests created a loophole for themes and the gap for a power interest matrix. A power interest matrix is mandatory for the analysis of diverse stakeholders. Newcombe (2003) elaborates that a power interest matrix is necessary for the classification of stakeholders based on their power and level of interest in the project [74]. A stakeholder grid was applicable post-data collection as all the perceptions and involvement of stakeholders were analysed and coded accordingly.

### 5.5. Qualitative Interviews

The primary instrument for stakeholder engagement/data collection was the utilisation of semi-structured questionnaires (Appendix A). Semi-structured questionnaires are effective for structuring the interview process, in addition to creating loopholes for emerging themes and topics initially not identified by the researcher [41]. Mack (2005) (p. 4) concurs that semi-structured questionnaires “allow greater spontaneity and adaptation of the interaction between the researcher and the study participant” [75].

Open-ended questions dominated the questionnaire, allowing the dialogue to extend and potentially create the opportunity for the emergence of additional themes and data not covered by the questionnaire [75]. Semi-structured questionnaires can be time-consuming but produce substantial results and discussions. Semi-structured interviews are relevant in the study, as they allowed the researcher and stakeholders to critically engage in dialogue pertaining to the traditional medicine sector and its legal implications.

The study received ethics approval from the University of KwaZulu-Natal’s Institutional Review Board (Approval number: HSS/0707/018M). Informed consent was obtained from all participants prior to all interviews.

### 5.6. Secondary Data

Secondary data was obtained through a variety of search strategies. The utilisation of Google scholar was the primary search strategy that led to the discovery of additional academic websites and subsequent journals. Secondary data consists of existing data that has previously been collected by another researcher usually from a different research question [76].

### 5.7. Coding

The coding of primary data was of vital importance to the study. “Coding is the process of analysing qualitative text by taking them apart to see what they yield before putting the data back together in a meaningful way” [77,78] (p. 15) and (p. 2850) respectively. Bryman and Burgess (1994) cited in Elliot (2018) assert that there is a considerable amount of confusion when it comes to understanding and applying coding [78]. They argue that it is doubtful whether writers who employ the term are referring to the same procedure [78]. Coding is context-specific and therefore allows each study to have a unique coding system dependant on various factors such as sample size, data size as well as frequency. For this particular study, data obtained from stakeholder interviews was categorised into alphabetical themes.

Transcriptions of stakeholder interviews were printed and highlighted according to the themes of categories. Coding can be a way of tagging data that is relevant to a particular point- for example identifying all the places in an interview where a stakeholder states something relevant to a particular question [78]. From the analysis of the interviews with key stakeholders, core themes were identified and were used to code the data obtained from the stakeholder interviews. It must be considered that certain responses overlapped and could be applicable to various themes. The themes however allowed for the systematic analysis of data.

### 5.8. Thematic Analysis

The study adopts a thematic method of analysis. A thematic analysis is appropriate for the study as the perceptions and varying roles and involvement of stakeholders are coded into different themes. Thematic analysis involves identifying, analysing, and reporting patterns within data [79,80]. The identification of common threads that extend across an interview or interviews is the essence of the thematic analysis method [80,81]. From the analysis of the recorded and transcribed stakeholder interviews, four common themes and threads emerged namely Stakeholders and the environment: perceptions and involvement, Education and awareness, Stakeholder roles and capacities, and Challenges. Thematic analysis of data is relevant to the study as the paradox between the traditional medicine sector and biodiversity conservation could be contextualised across different institutions represented by the interviewed stakeholders.

## 6. Results and Discussion

### 6.1. Stakeholders and the Environment: Perceptions and Involvement

Stakeholders play a significant role in biodiversity conservation and the overall sustainable development of South Africa [82]. Stakeholders in various organisations encounter a diversity of challenges in their attempt to adhere to the sustainable development agenda. The experiences and encounters of stakeholders subsequently shape their perceptions of South Africa’s natural environment and the sectors that emerge from it. The traditional medicine system is one that cannot be exempted from the biodiversity conservation sector [83]. The study’s primary intention was to evaluate the involvement of stakeholders and the legal instruments used to enforce existing environmental legislation in the traditional medicine system.

“*We do not have a problem with people using the resources if it is in a sustainable manner. We are mandated to look after the indigenous flora and fauna of the province and that is why we have got legislation in place. People need to ask questions and we will gladly assist*”.

(Stakeholder 1: 25 February 2020)

It is evident that there is a gap between stakeholders and those involved in the gathering, harvesting, and selling of medicinal species [84]. The gap is primarily widened by the lack of knowledge and understanding of environmental legislation. If THPs and communities have sufficient knowledge and understanding of the biodiversity sector, a range of biodiversity threats will be alleviated.

“*Legislation allows for the selling of protected and specially protected species. It is not prohibited. The key is sustainable usage*”.

(Stakeholder 1: 25 February 2020)

“*As I have observed over the years, most species sold are just protected species. It’s the more common and available, although recently plant material is becoming very scarce, even THPs are finding it very difficult to find specific species that their clients are requiring*”.

(Stakeholder 1: 25 February 2020)

From a legal perspective, THPs are misinformed on the species that are protected and those that are specially protected. Selling of both protected and specially protected species requires a licence. The permitting system is used to control and monitor species utilisation and therefore THPs are obligated to indicate the type of species they require. The key is not to restrict THPs, but to control, monitor and conserve species.

“*When it comes to just the protected plant material, the law doesn’t prescribe that a permit is required to gather plants but what is needed is -if you go on private property or state property /municipal property is a letter of authority from the landowner to go and gather*”.

(Stakeholder 1: 25 February 2020)

“*We monitor the threats to biodiversity by recording incidents of illegal collecting and poaching. We have a database. We monitor certain species that we know are used for traditional health practices and other traditional uses. For example, vultures are monitored to keep track of their availability status*”.

(Stakeholder 1: 25 February 2020)

“*Some species of plants and animals require a permit as they are categorised under the TOPS regulations. Therefore, traditional healers/traders at herb markets are required to produce permits to check whether they comply. They are further requested to produce a standing permit which allows for continuous carrying out of restricted activity in terms of the NEMBA Act No. 10 of 2004*”.

(Stakeholder 2: 30 September 2019)

“*THPs at Muthi markets are asked to produce identification and their stock is checked against the permitted items list. Items are ceased if not part of the list and the person selling is charged or given a warning. We do not issue compliance notices as they are not effective*”.

(Stakeholder 1: 25 February 2020)

“*We have observed a great amount of waste found in the various Muthi markets. Waste management is part of our consideration and we know THPs do not sell everything and there’s a very high volume of wastage and that concerns us*”.

(Stakeholder 1: 25 February 2020)

“*We have got provincial legislation which we are mandated to enforce. The Nature Conservation Ordinance 15 of 1974*”.

(Stakeholder 1: 25 February 2020)

Evidently, stakeholders carry out their operations and enforcement strategies based on their legal mandate. There is a distinction amongst the interviewed stakeholders. This, therefore, gives room for a diversity of legal instruments within the biodiversity conservation sector. Irrespective of the diversity of legal instruments, the sole purpose is to ensure that THPs do not exacerbate biodiversity loss.

“*There has to be a political will to ensure that our province’s biodiversity is preserved. If the municipality is looking for votes prior to elections the last thing they are going to do is go and cause trouble at these Muthi markets, because people will turn against them. And what is unfortunate is that it happens throughout the province and that is why we need to have the political will to ensure that the province’s biodiversity remains available to everyone and not just the select few*”.

(Stakeholder 1: 25 February 2020)

As stated by one of the stakeholders, there is a range of factors that contribute to the sustainability of the environment. Politics also play a crucial role in the level of compliance from THPs and communities in general. Politics is synonymous with incentives. People are most likely to adhere to and comply with legislation and restrictions if there are incentives involved [85].

“*If there were incentives and funding involved in the conservation of the environment, people would be alert and motivated to comply. For example, people live close to nature reserves but do not benefit from them, thus they are not concerned about any issues affecting the nature reserve*”.

(Stakeholder 4: 18 December 2019)

“*In addition, their sense of compliance is influenced by their indigenous knowledge. A traditional leader will instruct the community members to not harvest a particular plant at a certain period because of the weather conditions during that period. There is a belief that if you harvest at a particular time, it will anger the ancestors and the weather conditions will be unfavourable*”.

(Stakeholder 4: 18 December 2019)

The biggest challenge in the traditional medicine sector and the overall biodiversity conservation system is that of attempting to transition generational mindsets. The existence of Indigenous Knowledge Systems (IKS) cannot be exempted from the factors that contribute to peoples’ (including THP’s) resistance to change. Irrespective of regulations and policies, THPs practice according to their beliefs and the demands of the traditional medicine market.

“*It comes down to money. For example, if your permit allows you to sell 120 Kilograms of Cycad bark and you can get away with two, you’ve made a bigger profit*”.

(Stakeholder 1: 25 February 2020)

From the stakeholder perceptions, it is clear that there have been substantial efforts towards biodiversity conservation and the alleviation of exploitative practices such as overharvesting and gathering of medicinal species in large quantities. The enforcement of environmental legislation in Muthi markets has the potential to decrease the high number of species sold illegally as well as the number of THPs operating without the required permits. Table 6 illustrates the primary stakeholders interviewed that are involved in the enforcement procedures that take place at Muthi markets.

There are considerable factors that impact the level of compliance from THPs. Compliance may be due to the prior engagement that takes place. Although prior engagement has its setbacks it is important as all stakeholders should be given the opportunity to comment and provide their input into the development of decisions that affect them. This is crucial as we see resistance from traders where there is a lack of prior engagement.

If traders are not actively sought out in the long run, they may demand to be consulted. Problematic situations could arise when traders do not actively engage but are forced to do so by the demands of other stakeholders as a result of a crisis situation. In response, organisations may exercise crisis management techniques which often result in a forced, defensive dialogue between stakeholders. This kind of antagonistic interaction can be detrimental as it can create a substantial and enduring loss of reputation as well as damage trust between organisations and stakeholders.

There is an inherent need for meaningful stakeholder engagement where there is a willingness to change. Meaningful stakeholder engagement entails interaction, encouragement, inclusivity, and preparedness for change. “Engagement should be regarded as any other business project planning process, with adequate analysis, preparation, implementation, reporting, evaluation and follow up” [86] (p. 15). “The ideal stakeholder engagement process should be an iterative process, allowing engagement to benefit from diligent planning, thorough reporting, and the application of learning as a result of appropriate evaluation and monitoring” [86] (p. 15). Jeffery (2009) further proposes a seven-stage cycle that indicates the process flow of stakeholder engagement [86].

▪ Stage 1: Plan
▪ Stage 2: Understand
▪ Stage 3: Internal preparation and alignment
▪ Stage 4: Build trust
▪ Stage 5: Consult
▪ Stage 6: Respond and implement
▪ Stage 7: Monitor, evaluate and document

Following the proposed steps can potentially lead to successful stakeholder engagement. It is crucial for stakeholders to understand and trust each other therefore resulting in improved relationships and the overall implementation of projects and compliance with relevant policies or legislation. Consultation is crucial as it allows for constant understanding between stakeholders. Monitoring, evaluating, and documenting are paramount for keeping stakeholders updated and informed about the processes and changes that occur. It cannot be ignored that stakeholder engagement has its challenges.

**Table 6:** Stakeholders primarily involved in environmental law enforcement.

	Organisation 1	Organisation 2
	Custodians for the Environment	Policy and Lawmakers
Roles and enforcement practices		
Awareness Campaigns	No prior Engagement	Prior Engagement
Herb Market Raids	Unsuccessful: Resistance from traders.	Successful: Compliance by traders.
Other Stakeholders	Metro Police eThekwini Municipality	Metro Police SAPS
Law Enforcement	Warnings recorded and signed by offenders Compliance notices not issued.	Prosecution: Fines and Imprisonment

### 6.2. Education and Awareness towards Biodiversity Conservation and the Curbing of Exploitative Practices within the Traditional Medicine Sector

Effective education and awareness initiatives have the potential to modify and improve the mindsets of society [87]. A majority of the interviewed stakeholders attributed the exploitation of flora and fauna for medicinal purposes to the lack of knowledge and awareness pertaining to the environment. The implementation of education and awareness initiatives ensures that people understand the value of biodiversity and what steps need to be taken toward protecting the environment [88]. The interviewed organisations are however mandated to educate and promote awareness of biodiversity conservation. There are stakeholders that are responsible for ensuring that the mandate of education and awareness within the respective organisations are adhered to. For some organisations, there are units strictly responsible for education and awareness.

“*Awareness campaigns are conducted at schools and to THPs. Principles of sustainable utilisation of resources are emphasized*”.

(Stakeholder 1: 25 February 2020)

Education and awareness initiatives ultimately create platforms for THPs and communities to be trained and taught the basics of biodiversity conservation, including legislation. In some regions, communities are taught how to fill in permit application forms. The basic training in filling in application forms has the potential of reducing the number of people that avoid applying for permits as they cannot fill them in or understand the requirements or the language.

“*A district conservation officer in Jozini processes about 1200 applications once a year. He meets with the gatherers and THPs, where they are provided with application forms and are assisted to fill them amongst other things. And at the same time, they are given an awareness discussion by the district conservation officer*”.

(Stakeholder 1: 25 February 2020)

“*Education and training department facilitates programmes such as HIV/AIDS counselling and testing training as well as programmes for designing referral forms*”.

(Stakeholder 5: 6 January 2020)

Education and training for THPs make them well-equipped to deal with healthcare issues such as the HIV/AIDS pandemic. According to Stakeholder 4, a referral form is a document that states that an individual consulted with a traditional health practitioner and is being referred to a hospital.

“*The referral form system signifies the possibility of a collaboration of traditional healthcare with primary healthcare. Referral forms are however not common and not recognised by some hospitals*”.

(Stakeholder 5, 6 January 2020)

“*There is currently no policy regulating the collaboration of THPs with primary healthcare in South Africa*”.

(Stakeholder 5: 6 January 2020)

Education and awareness from a legislative perspective, are conducted to promote compliance by THPs. For some organisations, awareness campaigns are conducted prior to enforcement strategies such as the raiding of herb markets.

“*…This gives an opportunity to establish any challenges that are faced between the involved parties or an opportunity to get a clear understanding of environmental legislation that regulates THPs. After which they are given a period of time (an estimate 2/3 years) to apply for a permit*”.

(Stakeholder 2: 30 September 2019)

“*There are awareness campaigns which are held not to just educate THPs but also the general public about the environment. The department also takes part in events such as career expos and school visits to promote the conservation of biodiversity*”.

(Stakeholder 2: 30 September 2019)

The consensus amongst the stakeholders was that of the importance of education and awareness towards curbing biodiversity loss, excessive harvesting of medicinal plant species as well as the significance of promoting environmental awareness in schools and communities. Mohamed et al. (2006) [89] (p. 1) elaborate that environmental education and awareness “increase human capacity to participate in environmental management and in solving crisis and challenges”. Education and awareness campaigns subsequently evoke a sense of stewardship and responsibility within the affected parties or communities Furthermore, biodiversity stewardship initiatives are potentially the most efficient way to promote conservation and sustainable practices within communities and the traditional medicine sector. With effective education and awareness, THPs would harvest and gather medicinal plant and animal species in sustainable and moderate quantities and drastically reduce the threats to the availability of vital flora and fauna.

### 6.3. Stakeholder Interests, Roles, and Capacities

It is of vital importance to analyse and understand the various stakeholder roles within the biodiversity sector. The roles of stakeholders determine their power and influence and subsequent decision-making capacities [42,87]. The study comprised of stakeholders in various capacities with the sole purpose of conserving the environment and promoting the enforcement of current environmental legislation.

“*We assist law enforcement bodies to identify the threatened or protected species and if THPs are in possession of them—we require documentation (permits) allowing them to be in possession and trading TOPS species. If not, we cease the specimen and hand it over to the enforcement team*”.

(Stakeholder 2: 30 September 2019)

The South African government has implemented numerous strategies for curbing biodiversity exploitation. Amongst these initiatives, the then Department of Environmental Affairs (DEA) released a Compliance report for the years 2017–2018. Amongst their various programmes, an awareness campaign initiative was introduced to the traditional medicine sector.

“*DEA together with provincial conservation departments had been receiving increased complaints from the public about a diverse number of threatened species listed in terms of the Threatened or Protected Species (TOPS) Regulations being illegally sold at Muthi markets across the country. It was important to ensure that the traders were aware of the legislation that protects these listed species, and accordingly, a decision was made that a proper awareness programme should precede compliance and enforcement activities in relation to this sector*”.

[78] (p. 75)

Initiatives ultimately reflect the interests and roles of organisations within the conservation sector. There are differing capacities at which these roles and interests can be projected. An organisation such as DEA (currently known as DEFF) has the capacity to implement initiatives at both local and national levels, subsequently making it a more powerful stakeholder. Organisations with lower capacities can however adopt similar initiatives at their designated scales. Politics also play a crucial role in the roles and capacities of stakeholders. Political ecology is therefore evident in the capacities and decision-making powers of organisations. A stakeholder grid is mandatory in the understanding of stakeholder roles, interests, and decision-making capacities.

“*We interact with traditional authorities and under traditional authorities, you find your THPs and general people who have knowledge of traditional medicine*”.

(Stakeholder 4: 18 December 2019)

“*When visiting communities, we first identify committees representing them, then we liaise with the committee for all proceedings pertaining to the community and our functions*”.

(Stakeholder 4: 18 December 2019)

“*We capacitate that committee to facilitate the activities and permits. If there are employment opportunities, individuals from vulnerable households are given first preference*”.

(Stakeholder 4: 18 December 2019)

“*Our core function is to facilitate THPs with primary healthcare in South Africa*”.

(Stakeholder 4: 6 January 2020)

**Figure 4:**
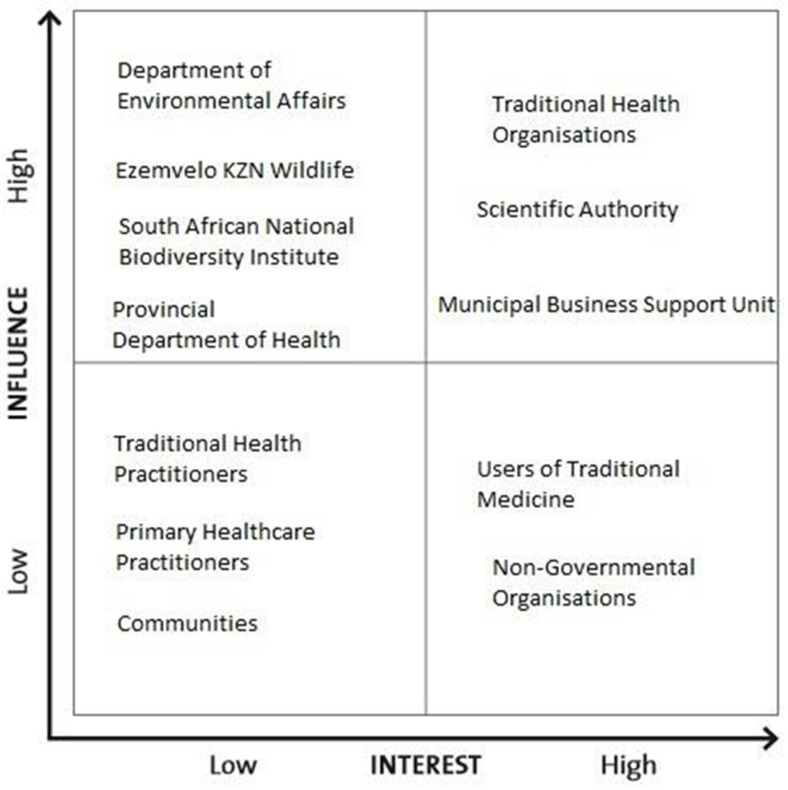
Stakeholder grid, using biodiversity conservation stakeholders and their interaction with the traditional medicine sector.

There are varying levels of interest and influence amongst the stakeholders involved in biodiversity conservation and its interaction with the traditional medicine sector as depicted in Figure 4. Vogler et al., 2017 define stakeholder grids as tools that can be used to visualise stakeholder influence and interests on a set of axes. Stakeholder grids help with the identification of potential group coalitions [40]. Stakeholders in the upper left axis, have the highest level of influence pertaining to the nexus between biodiversity conservation and the traditional medicine sector. These stakeholders influence legislation, licensing and the potential regulation of the traditional medicine sector based on sustainable grounds. Those in the lower-left axis, have less influence in terms of decision-making and law enforcement. Contrastingly, the stakeholders in the upper right axis have a greater interest in the traditional medicine trade, whilst users of traditional medicine and non-governmental organisations have minimal interest in the regulation and legal aspects of the trade in medicinal species. The stakeholder grid articulates that stakeholders may not necessarily share the same interests or opinions but are equally relevant to the accomplishment of a single project.

### 6.4. Challenges

Stakeholders in the conservation sector including law enforcement personnel, are faced with a variety of challenges in their attempts to enforce and conserve vital flora and fauna. Lack of compliance; hostility towards officers as well as the lack of cooperation from relevant departments, is among the many challenges listed by the interviewed stakeholders. A majority of the stakeholders stated that there is a general lack of trust and cooperation between officials and THPs. For some organisations, hostility and lack of cooperation are not challenges that they are faced with. From an environmental conservation perspective, illegal trading and gathering of species is an alarming challenge for some stakeholders. There is a need for stricter policies that control access and trading of medicinal plant species in herb markets.

“*We find Buckie and truckloads of plant material coming down from up North of KwaZulu-Natal or from the Eastern Cape, Gauteng and down to Durban. These people are transporting it, they are gatherers, but we know that they are going to bring it down here to sell to the THPs. So, in fact, the gatherers themselves should have a licence to sell the material, but they don’t and that is one of the gaps that we have basically got in legislation*”.

(Stakeholder 1: 25 February 2020)

In retrospect, it could potentially be that the people that contribute the most to biodiversity loss and overall environmental exploitation, are the gatherers of medicinal plant material. This could be due to the demand in supply from the THPs that they sell to, or alternatively, they could be selling to other provinces and neighbouring countries. One stakeholder mentioned that the municipality does not play a substantial role in controlling and monitoring species that are brought into the Muthi market.

“*The Municipality does not monitor the quantity of plants brought into the market. Anyone can drop off plant material. A THP will tell you that ‘I have a permit from the municipality to sell, so who are you to tell me about my stock?’. The lack of collaboration makes it very challenging to enforce legislation at Muthi markets. We find large quantities of unused medicinal plants and animals*”.

(Stakeholder 1: 25 February 2020)

“*There is a high level of animosity towards anybody in a uniform. Be it municipal, KZN Wildlife or the South African Police Services. One of the biggest problems is the prevention of ensuring compliance. You can’t just get in the market they chase you out*”.

(Stakeholder 1:25 February 2020)

One of the stakeholders highlighted that their duties as an organisation go beyond Muthi markets and into community-based initiatives. The organisations’ biodiversity stewardship programme allows communities involved in the utilisation of traditional medicine to be sustainable and aware of their practices. There are, however, inevitable challenges and conflicts. Amongst these challenges, the erosion of indigenous knowledge and lack of documentation is one that worries the organisation the most. Furthermore, the lack of interest from the youth is alarming and threatens the possibility of sustainable development in vulnerable communities.

“*In addition to the many challenges we encounter, we find people going into rural areas to harvest for the sake of selling. Their harvesting practices are unsustainable and profit-motivated. A rural person is more conservative of the environment compared to urban dwellers. Indigenous knowledge systems are what makes rural people more considerate of the environment*”.

(Stakeholder 4: 18 December 2019)

“*Deep rural areas feel very neglected. Researchers do not give feedback to communities, which makes them feel neglected and exploited for their knowledge*”.

(Stakeholder 3: 14 November 2019).

A common trend amongst the interviewed stakeholders is that of hostility and conflict from THPs. One of the stakeholders mentioned that many THPs are illiterate and struggle with interpreting and reading legislative material such as permits. Another stakeholder did, however, mention that their organisation’s education and awareness initiatives prioritise the training and teaching of legislative material amongst other sections. The language barrier between THPs and officials is very limited as a majority of District conservation officers can speak vernacular languages. It can, therefore, be considered that THPs use the language excuse as a way to justify their non-compliance.

“*We have observed that there is a heterogenous attitude from the THPs. They are very sceptical and refuse to share information that could potentially help us in improving and ultimately regulating their system*”.

(Stakeholder 5: 6 January 2020)

Challenges within the traditional medicine sector are inevitable. From the interviewed stakeholders, it is clear that there still needs to be great adjustments and enforcement strategies that will ultimately bridge the gap between the knowledge of stakeholders and THPs. There have been, however, substantial efforts toward enforcing conservation policies.

### 6.5. Political Ecology and Conservation Stakeholders

There is relevancy in placing the traditional medicine sector and its relationship with conservation policies in a political ecology context. Political ecology highlights the interconnections between the natural environment and socioeconomic implications motivated by political dynamics. The study focused on two primary stakeholders (national bodies of Biodiversity conservation and Traditional Health Practitioners) and their subsequent stakeholders. The political ecology theory is particularly relevant to this study because it provides the theoretical background required for understanding the existing and emerging conflict between government stakeholders and THPs. The integration of political ecology into the study justifies and contextualises the stakeholder perceptions and involvement in the biodiversity conservation sector.

To remedy the existing antagonism between THPs and environmental conservation officials, there is a need for strategies aimed at improving stakeholder engagements and overall relationships. Improved stakeholder engagements will result in greater understanding amongst stakeholders. There is a significant gap in research pertaining to stakeholder conflict management and successful engagements.

## 7. Conclusions

The paradox between biodiversity conservation and livelihood sustenance is reflected in the relationship between stakeholders and THPs. The contrasting knowledge systems, therefore, result in a hostile attitude from THPs, as environmental enforcement limits the excessive harvesting, gathering, and selling of medicinal plant and animal species. From a global perspective, the Convention on Biological Diversity (CBD) is the overarching treaty. In South Africa, the KwaZulu-Natal Nature Conservation Ordinance No. 15 of 1974 is one of the primary legal mandates used by stakeholders to control and regulate the harvesting and selling of traditional medicinal plant and animal species in the province. A mixed-methods approach lays the foundation of the study, as both qualitative and quantitative approaches are adopted in the study. In 2020, five prominent stakeholders within the biodiversity conservation sector were interviewed using the purposive sampling technique. The selected stakeholders represented various organisations involved in the nexus between traditional medicine and biodiversity conservation. For the elimination of conflict, the stakeholders and their subsequent organisations have been kept anonymous, thus an objective and non-bias representation of the organisations. The stakeholder analysis framework and political ecology theory are underlying frameworks that contextualise the relationship between contemporary environmental legislation and monetary livelihoods in South Africa. Stakeholders play a vital role in decision-making and implementation of projects and policies pertaining to biodiversity conservation. The decisions and interests of stakeholders characterise a political ecology. Changing the generational mindsets of THPs is a challenge that requires stringent law enforcement and compliance motivating strategies. Towards the promotion of compliance, incentives are highly recommended. The incentive approach to biodiversity conservation promotes compliance and environmental stewardship within communities that utilise medicinal plant and animal species. Stakeholders in the biodiversity conservation sector, have various roles and capacities that ensure that legislation is implemented, and trading of medicinal species are conducted on sustainable grounds. There is apparent evidence, that both the knowledge of conservation stakeholders and THPs are vital for the sustainable conservation of natural resources whilst sustaining monetary livelihoods. Amongst a range of challenges, the study found that there is a significant amount of conflict and hostility from THPs towards biodiversity stakeholders due to the lack of education and awareness initiatives; municipal officials do not co-operate with environmental enforcement officials thus creating challenges with enforcement and implementation of environmental mandates.

## Figures and Tables

**Table 2:** Compatible concepts within legal mandates.

Concept	Definition
Endangered Species	Indigenous species facing a high risk of extinction in the wild in the near future, although they are not critically endangered species
Vulnerable Species	Indigenous species facing a high risk of extinction in the wild in the medium-term future, although they are not critically endangered species
Critically endangered species	Indigenous species facing an extremely high risk of extinction in the wild in the immediate future.
Protected Species	Indigenous species of high conservation value or National importance that require national protection.
Specially protected species	Species of special conservation interest, migratory species or species subject to other international agreements.
Gather	To pick, pluck uproot, cut, accumulate, collect, cultivate, amass, chop off, saw off, break, or damage or destroy, whether wholly or partially.
Registered Wildlife trader	A person who may hawk, peddle, barter, exchange, offer, advertise, expose, or have in his or her possession for the purpose of exhibition, display, sale, hawking, peddling, bartering, or exchanging, any listed threatened or protected species, and includes taxidermists and game capturers;

Sources: [30,38,39].

**Table 4:** KZN Nature Conservation Ordinance no. 15 of 1974, and its relevant chapters, sections and conditions pertaining to the traditional medicine system. Source: United Nations Office on Drugs and Crime, 1974 cited in [70].

Section/Chapter	Conditions
Section 27: Board may appoint officers, honorary officers, and employees to enforce laws relating to fauna and flora outside reserves.	(1) The board may appoint such officers, honorary officers and employees as it may deem necessary for the proper and efficient administration of chapters II to XII and of any of the laws contemplated by Section 24(1), confer upon them the titles or designations by which they shall be known and determine their respective functions, powers and duties.
Chapter VII: Amphibians, Invertebrates, and reptiles Section 101: Kill or capture	(1) No person shall kill or capture any protected indigenous amphibian, invertebrate or reptile, save in accordance with a permit issued in terms of Section 106.
Chapter XI: Indigenous Plants Section 195: Permit to sell protected indigenous plants	(1) A protected indigenous plant may be sold only under the authority of a permit issued by the Board subject to such conditions as the board may impose.
Section 196: Licence to sell specially protected indigenous plants	(1) Specially protected indigenous plants may only be sold under the authority of and in accordance with a licence issued in terms of this section
Section 200: Gathering of specially protected indigenous plants	Subject to the provisions of Sections 196, 198 and 201, no person shall gather any specially protected indigenous plant save under the authority of and in accordance with a permit issued by the Board with the prior approval of the administrator, and such gathering shall only take place in the land, by the owner of such land, or by any person with the prior written permission of such owner, which permission shall at all times during such gathering be in the possession of such person and available for inspection immediately on request by an officer, honorary officer or employee of the board: Provided that the approval of the administration may be refused or granted subject to such conditions as the administrator may determine.
Section 202: Prohibition of gathering on public roads	Save as is provided by subsection (2), no person who is not in possession of a permit issued in terms of Section 200 or 201, shall gather any indigenous plant on any public road or in the road reserve of any public road without the prior permission of the administrator. Subsection (1) shall only apply in respect of indigenous plants unavoidably destroyed in the course of lawful road development or maintenance.
Section 204: Applications for and issuance of permits and licences	(1) Any permit of licence referred to in this chapter shall be issuable by an officer or other person authorised by the board for that purpose. (3) Every permit or licence issued in terms of this chapter shall be personal to the holder to whom it was issued and shall not be transferable to any other person.
Section 207:	Any person who falsifies or misuses any permit or licence to gather, sell or export or import indigenous plants shall be guilty of an offence.
Chapter XII: General Section 212: Powers of interest	Any officer, honorary officer or employee of the Board thereto authorised by the Administration shall have the power to arrest without a warrant any person suspected upon reasonable grounds of having contravened any provision of this ordinance: Provided that no officer or employee so authorised as aforesaid shall arrest any person without a warrant unless he has reason to believe that the ends of justice will be defeated by the delay in obtaining a warrant

**Table 5:** Stakeholder codes, interests, and influences.

Stakeholder Code	Roles/Interest	Influence/Power
1	Biodiversity conservation Environmental Law Enforcement	Legislation Permit issuing
2	Sustainable development Environmental Education	Law enforcement (EMI) Herb market inspections
3	Facilitation of THPs in eThekwini	Provincial decision making Provision of THPs for research purposes
4	Environmental stewardship initiatives	Community education and awareness initiatives Incentive-based conservation
5	Regulation and facilitation of traditional health care	Provincial policy implementation Registering and facilitation of THPs

## Appendix A. Stakeholder Questionnaire

**Whose Knowledge? Examining the Relationship between the Traditional Medicine Sector and Environmental Conservation Using a Stakeholder Analysis: Perceptions on Warwick Herb Market Durban South Africa**

Stakeholder Questionnaire

- NB. The participant is at liberty to withdraw from the survey at any time, should they desire.
- Responses will be typed in a separate sheet.

**Background Questions**

1. What is your role as a stakeholder in your Organisation?
2. Does your line of work include dealing with traditional health practitioners? If yes, please elaborate.
3. As a nature conservation organisation, how does the unsustainable harvesting and hunting of wildlife for medicinal purposes affect the environment and biodiversity?
4. As a stakeholder, what are the biggest challenges when it comes to traditional medicine and environmental conservation?
5. What measures are in place to control the exploitation of vital flora and fauna due to the increasing demand for traditional medicine?
6. Can you name some of the species prone to exploitation? Are they listed as protected, endangered, or threatened?
7. How does your organisation ensure that traditional health practitioners are compliant with relevant environmental legislation/conservation policies?
8. Are there any existing programmes/initiatives that are for educating traditional health practitioners and the general public about the environment, environmental effects of wildlife trading?

**Section A: Law Enforcement and compliance in Herb Markets.**

1. Does your organisation have a section/department that is specifically responsible for activities associated with traditional medicine?
2. If yes, what is the name of the section/department? And what are the core functions of the section/department?
3. How does your organisation ensure that Traditional Health Practitioners are compliant with the relevant environmental legislation/policies?
4. What procedure is used to check for compliance in herb markets?
5. Is there any form of engagement with Traditional Health Practitioners prior to the raids that take place in Herb Markets?
6. What are the legal actions taken against offenders?
7. Which other stakeholders/Departments are involved in the operations used to check for compliance and what are their roles?
8. What are the common challenges encountered in Herb Markets? How are they dealt with?
9. What measures are in place to control the exploitation of vital flora and fauna due to the increasing economic demand for traditional medicine?
10. What is your take on contemporary conservation policies and their influence on monetary livelihoods?
11. Are there any conservation interventions currently implemented by your department/organisation? If yes, how have they impacted vulnerable communities (e.g., Communities depending on natural resources as a primary source of livelihoods).

**Section B: Environmental Education and Awareness; Biodiversity Conservation.**

12. Are there any existing programmes/initiatives that are for educating Traditional Health Practitioners and the general public about the environment; the environmental effects of wildlife trading; and the dangers of exploiting vital flora and fauna? If not, are there plans in place to introduce these topics in future?
13. As a mitigation strategy, is there a plan for curbing wildlife poaching; overharvesting and illegal wildlife trading?
14. How does your department/organisation monitor the threats to biodiversity? Which policies are in place to ensure the sustainability of vital flora and fauna in South Africa?
15. Are there existing research studies currently being undertaken by your department/organisation that are specifically on the link between conservation policies and monetary livelihood systems?
16. Are you involved in the formulation of conservation policies? If yes, what are the general requirements in formulating such policies?

**Section C: General perceptions on the traditional medicine sector /monetary livelihood systems.**

17. In your opinion, do you see the possibility of the integration of traditional medicine into primary healthcare? Why?
18. The traditional medicine sector is said to have a staggering market value, do you see its potential towards contributing to economic growth and development in South Africa? Why?
19. With effective education and training, do you see Traditional Health Practitioners changing the dynamics of the South African health system?
20. What is your opinion on Indigenous Knowledge Systems and their relevance in the current sustainable development agenda?
21. Is there an existing database of all the traditional medicine and their common names and uses? If yes, where can one obtain it or have access to it?
